# COVID-19: a potential driver of immune-mediated breast cancer recurrence?

**DOI:** 10.1186/s13058-020-01360-0

**Published:** 2020-10-30

**Authors:** Federica Francescangeli, Maria Laura De Angelis, Ann Zeuner

**Affiliations:** Department of Oncology and Molecular Medicine, Istituto Superiore di Sanità, Rome, Italy

**Keywords:** COVID-19, Breast cancer, Dormancy, Metastasis, Relapse, Inflammation, Disseminated tumor cells, Tumor recurrence, Metastatic reawakening

## Abstract

Severe coronavirus disease 2019 (COVID-19) causes a hyperactivation of immune cells, resulting in lung inflammation. Recent studies showed that COVID-19 induces the production of factors previously implicated in the reawakening of dormant breast cancer cells such as neutrophil extracellular traps (NETs). The presence of NETs and of a pro-inflammatory microenvironment may therefore promote breast cancer reactivation, increasing the risk of pulmonary metastasis. Further studies will be required to confirm the link between COVID-19 and cancer recurrence. However, an increased awareness on the potential risks for breast cancer patients with COVID-19 may lead to improved treatment strategies to prevent metastatic relapse.

Severe acute respiratory syndrome coronavirus 2 (SARS-CoV-2) pandemic is spreading in a world where cancer prevalence is rapidly growing, raising concerns about potential interactions between the two diseases. SARS-CoV-2 recruits proteins involved in cellular replication, DNA damage, metabolism, and epigenetic regulation that are also implicated in cancer pathogenesis [1]. At the same time, COVID-19-induced inflammation may affect tumor cells and their microenvironment. The effects of COVID-19 on breast cancer are still unknown. However, emerging evidences suggest that COVID-19 may affect a particular stage in the tumor’s life cycle represented by dormant cancer cells (DCCs). DCCs often survive upon successful treatment of primary tumors and localize in specific microanatomical compartments of metastasis-prone organs, where they can reside in a quiescent state for a clinically asymptomatic period named metastatic dormancy [2]. At some point, DCCs may reactivate in response to microenvironmental cues such as inflammatory or immune-mediated signals, thereby progressing to overt metastasis. Virtually, every patient with a previous history of cancer may harbor DCCs. In breast cancer, understanding the mechanisms underlying cancer cell dormancy and reawakening is of crucial importance due to a particularly broad window of tumor recurrence, encompassing up to two decades after diagnosis.

SARS-CoV-2 infection induces the death of airway epithelial cells with consequent release of damage-associated molecular patterns (DAMPs). DAMPs trigger the production of inflammatory cytokines and chemokines, thus recruiting monocytes, neutrophils and T cells to the lungs (Fig. 1). In the severe phase of COVID-19, lung inflammation leads to diffuse alveolar damage and acute respiratory distress syndrome (ARDS). Moreover, activated immune cells can start a pro-inflammatory loop resulting in systemic inflammation, widespread coagulopathy, and multiorgan dysfunction. Profound immune system alterations also occur upon SARS-CoV-2 infection, including a decrease in natural killer cells and T cells in the peripheral blood, and a dysregulated activation of monocytes, neutrophils, and tissue macrophages [3]. Activated neutrophils release multiple tissue-damaging products including web-like structures of proteins and DNA known as neutrophil extracellular traps (NETs). NETs entrap pathogens and provide for a high local concentration of antimicrobial components, but also create a physical barrier that hinders local access to immune cells.

**Fig. 1:**
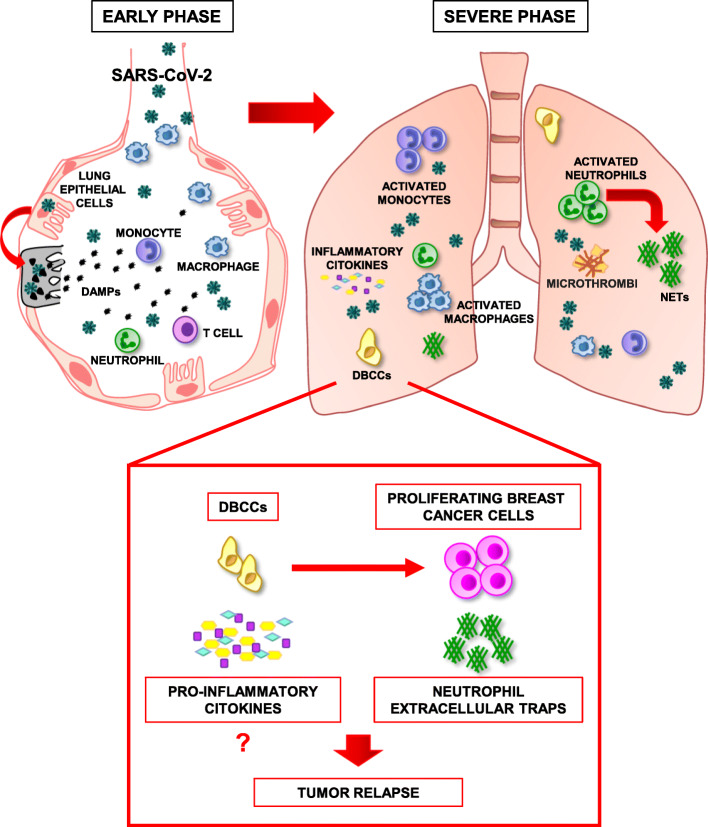
Modifications in the lung microenvironment occurring during the early and severe phases of SARS-CoV-2 infection and potentially involved in the reawakening of dormant breast cancer cells (DBCCs). During the early phase of COVID-19 (left), SARS-CoV-2 enters pulmonary alveoli and infects airway epithelial cells, which undergo cell death releasing damage-associated molecular patterns (DAMPs). DAMPs activate neighboring cells, starting an inflammatory response that in the severe phase of the disease (right) results in an overproduction of inflammatory cytokines and recruitment of activated monocytes, macrophages, and neutrophils. The latter produces neutrophil extracellular traps (NETs), which contribute to inflammation, immune escape, and thrombosis. NETs, and possibly pro-inflammatory cytokines, can cause DBCC reawakening leading to metastatic outgrowth and tumor relapse

Several factors involved in COVID-19 may play a role in the reawakening of dormant tumor cells. The strongest evidence points to NETs and neutrophils, which are emerging as important players in COVID-19 pathogenesis. NETs involvement in COVID-19 was first proposed upon observation of intense neutrophilic infiltration in the lungs of autopsied COVID-19 patients [4]. The presence of NETs in COVID-19 patients was then confirmed and showed to be responsible for immunothrombosis [5]. Acute lung inflammation and NETs have been respectively shown to trigger the exit from dormancy of breast DCCs, leading to metastasis formation [6, 7]. First, lung inflammation induced by bacterial lipopolysaccharide was shown to induce epithelial-to-mesenchymal transition (EMT) and metastatic reawakening in breast DCCs [7]. Secondly, laminin destruction by NET-associated proteases was reported to activate integrin signaling in lung-resident DCCs, thus inducing proliferation and lung metastasis [6]. Therefore, lung inflammation and NET generation that occur during COVID-19 could trigger DCCs reawakening, possibly acting in concert with other pro-inflammatory factors (Fig. 1). Among these, elevated levels of interleukin-6 and other pro-inflammatory cytokines released during severe COVID-19 result in a widespread activation of NF-κB in both immune and non-immune cells. The induction of NF-κB activation in pre-metastatic niches may contribute to DCCs reawakening both directly by stimulating cancer cell proliferation and indirectly by inducing the formation of a pro-metastatic microenvironment.

Hypoxia, which arises in the blood and tissues of COVID-19 patients upon respiratory distress and thrombosis, is a poor-prognosis microenvironmental hallmark of solid tumors. In breast cancer, hypoxia has been shown to be responsible for DCCs generation by promoting the expression of genes implicated in dormancy, drug resistance, stemness, and EMT [8]. Therefore, hypoxic microenvironments present in COVID-19 patients may play a double role on DCCs, on one side promoting dormancy but on the other side generating an aggressive drug-resistant phenotype that lays the ground for subsequent tumor relapse.

Finally, recent studies on the long-term clinical outcomes of COVID-19 showed a high incidence of persistent symptoms after the acute disease [9]. The possibility that inflammatory and/or autoimmune processes may be a common consequence of SARS-CoV-2 infection raises further concerns about the risks of DCCs reawakening, which may be enhanced in chronically inflamed microenvironments.

Ongoing clinical studies that include an assessment of the long-term effects of COVID-19 on cancer patients [10] will clarify the effects of COVID-19 on the risk of pulmonary metastatic recurrence. If confirmed, the association between COVID-19 and an increased risk of lung metastasis can promote the use of tailored therapies and intensified follow-up schedules in patients with a previous breast cancer. In particular, the use of anti-inflammatory agents able to interfere with immune-mediated inflammatory pathways or NET formation could be helpful in decreasing the risks of subsequent tumor relapse.

## Data Availability

Data sharing is not applicable to this article as no datasets were generated or analyzed during the current study.
